# A ‘Music, Mind and Movement’ Program for People With Dementia: Initial Evidence of Improved Cognition

**DOI:** 10.3389/fpsyg.2019.01435

**Published:** 2019-07-16

**Authors:** Olivia Brancatisano, Amee Baird, William Forde Thompson

**Affiliations:** ^1^Department of Psychology, Faculty of Human Sciences, Macquarie University, Sydney, NSW, Australia; ^2^Centre for Scaffolding the Ageing Mind, Faculty of Human Sciences, Macquarie University, Sydney, NSW, Australia

**Keywords:** music, movement, dementia, cognition, fluency, attention, therapeutic

## Abstract

**Background:**

Music is being increasingly used as a therapeutic tool for people with dementia. Research has uncovered several qualities of music that are responsible for its beneficial effects. Based on the identification of seven therapeutic capacities of music, we devised the *Music, Mind, and Movement* (MMM) program and evaluated whether it had therapeutic benefit for people with dementia (various types) in the areas of cognition, mood, identity, and motor fluency.

**Methods:**

The MMM program involved seven 45-min weekly group sessions, and individual 15-min “booster” sessions. Twenty people with mild to moderate dementia participated. Group 1 (*n* = 10) completed the MMM program first and Group 2 (*n* = 10) acted as a wait list control for 7 weeks, receiving standard care and completing the MMM program after the first group. Assessments of global cognition (Addenbrooke’s Cognitive Examination, ACE-III), mood (Geriatric Depression Scale short form), identity (‘I am’ task), and fine motor skills (9-Hole peg task) were conducted at baseline (T1), time 2 (T2, post treatment), and time 3 (T3, 1 month post MMM program).

**Results:**

Within group comparisons were conducted with 12 participants from the MMM program and 10 participants receiving standard care. Global cognition (total ACE-III score) improved in 8/12 participants after the MMM program, whilst it decreased in 8/10 participants after the period of standard care. MMM participants showed increases in ACE-III subdomain scores of attention (*p* = 0.007) and verbal fluency (*p* = 0.056).

**Conclusion:**

Our preliminary findings suggest that the MMM program may improve cognition, particularly verbal fluency and attention, in people with dementia.

## Introduction

Dementia is an umbrella term used to describe a group of neurodegenerative disorders that cause a decline in cognitive function, impacting on everyday skills. Currently, dementia affects approximately 50 million people worldwide (*Alzheimer’s Disease International*). The most common form of dementia is the Alzheimer’s type, accounting for approximately 70% of cases. The hallmark symptom of Alzheimer’s dementia (AD) is impaired memory. There is no cure for dementia, although certain pharmacological treatments can improve some symptoms (e.g., Howard et al., 2012). Nevertheless, many of these pharmacological treatments have side effects and are ineffective for some individuals. Therefore, there is a demand for non-pharmacological treatments, especially if such alternative therapies confer behavioral and psychological benefits that are equal to those observed for pharmacological therapies, without any of the adverse events (Dyer et al., 2018).

**FIGURE 1:**
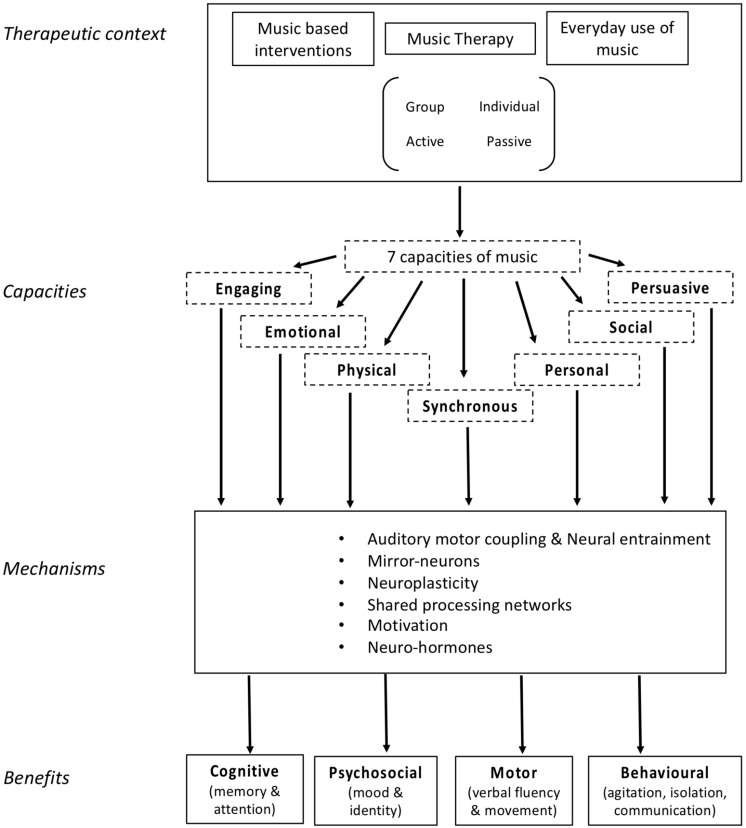
The Therapeutic Music Capacities Model (TMCM).

Music has been used as a therapeutic intervention for people with numerous neurological disorders, particularly in dementia care (for review, see Altenmüller and Schlaug, 2013; Thompson and Schlaug, 2015). It has been proposed that the therapeutic value of music may be attributed to seven distinct capacities of music. Namely, that music is persuasive, engaging, emotional, personal, physical, and social, and it affords synchronization (Thompson and Schlaug, 2015). Together, these capacities comprise a robust blend of affordances that can be used in a therapeutic setting to address many of the symptoms of dementia, such as memory decline, decreased language fluency, and an altered sense of self (Brancatisano and Thompson, in press). These capacities form the basis of the *Therapeutic Music Capacities Model* (TMCM, Figure 1), which outlines the capacities and the therapeutic outcomes that arise as a result of their therapeutic potential. We evaluated the efficacy of a newly developed music-based program, the Music, Mind, and Movement (MMM) program, which is based on this model.

There are three fundamental advantages of using music for therapy with people with dementia. Firstly, music is easy to access and deliver as therapy. Particularly with recent advances in technology, music is more ubiquitous than ever before. We have access to thousands of songs, spanning culture and time, in a range of settings, from individual music listening with iPods to group settings. This makes music suitable to the dementia population since individuals are able to partake in the experience (whether through listening, moving or music making) irrespective of their level of functioning. Furthermore, the negative side effects of such interventions are rare. Negative experiences of music can arise if the music is intrusive or otherwise unappealing (Chang et al., 2010; Nair et al., 2011), or if the music reinforces depressive tendencies (Garrido et al., 2017, 2018), but such negative effects tend to be transient and easily managed by removing the individual from the musical source.

Secondly, musical functions, including some forms of musical memory, are often spared in the face of AD, even during the most severe stage. Some of these preserved musical abilities include the detection of wrong notes in familiar songs (Cuddy and Duffin, 2005), learning new songs in both musicians with dementia (Cowles et al., 2003) and non-musicians with dementia (Prickett and Moore, 1991; Samson et al., 2009; Baird et al., 2017), the ability to detect emotional meaning in music (Drapeau et al., 2009) and show emotional responses to music such as joy (Norberg et al., 2003; Baird and Thompson, 2018). These observations of spared music abilities in the face of dementia opened the door to the possibility of using it as a means of therapy in dementia care.

Lastly, music can prime or scaffold other (non-musical) functions. For example, music can stimulate autobiographical memory (e.g., Irish et al., 2006; Baird et al., 2018). It also engages the individual in new learning, exercise and cognitive training, and thus can reinforce the processes of ‘neural scaffolding.’ This is a process, originally defined by Park and Reuter-Lorenz (2009) in their ‘Scaffolding Theory of Aging and Cognition’ (STAC) model, which explains how life-course factors can enhance or deplete neural resources, influencing the developmental course of cognition and brain function. The STAC model proposes that as individuals age, certain enriching factors may enable ‘compensatory scaffolding’ to occur, which may protect against cognitive decline. In the face of dementia, neuropathology may undermine the brain’s ability to provide effective compensation. Music and its broad network of capacities can engage brain regions that are involved in neural scaffolding, such as frontal areas that are relatively preserved in people with the most common type of dementia, AD.

In the last decade music-based activities have been implemented as a way of alleviating negative symptomology associated with dementia (primarily for AD), such as agitation (Raglio et al., 2010), anxiety (Sung et al., 2010, 2012) and depression (Ray and Mittelman, 2017). Further, cognitive function has been shown to improve during or immediately after music-based treatments (Van de Winckel et al., 2004; Chu et al., 2014; Särkämö et al., 2014). There have been two Cochrane reviews that have examined the effectiveness of music interventions for behavioral, emotional and cognitive outcomes in people with dementia (Vink et al., 2003; Van der Steen et al., 2017). Vink et al. (2003) included ten randomized control trials (RCTs) and the second, more recently, by Van der Steen et al. (2017) included 17 RCTs. Vink et al. (2003) stated that no conclusions could be drawn as the quality of methods was poor overall. The later 2017 study was able to conclude that the evidence was ‘moderately strong’ to support the use of music to improve symptoms of depression, but not agitation or aggression. Evidence that music can improve mood, cognition, anxiety and social interaction was low, with issues such as risk of bias and small sample sizes. Thus, whilst there is support for music as a treatment for certain symptoms of dementia, ambiguity exists surrounding its effectiveness for various other symptoms of dementia.

Part of the reason for this ambiguity may be the multitude of different ways music is used therapeutically. Music interventions range from traditional music therapy, an evidence-based practice involving a trained music therapist, to music-based programs led by a musician or facilitator with no music training. These music-based programs can involve listening to music (receptive) or music making (active), such as singing or using an instrument. The music used in these interventions can be researcher or participant chosen depending on the outcome desired, such as using personal music to promote reminiscence. In addition, these activities can be experienced either individually or in a group. Each of these modes of therapies has a variety of beneficial effects. Active music therapy in a group setting has been shown to improve general cognition, measured using the Mini-Mental State Examination (MMSE, Bruer et al., 2007; Chu et al., 2014), and specific cognition functions, such as verbal fluency (Brotons and Koger, 2000; Lyu et al., 2018). Group music therapy has also demonstrated benefits by reducing associated symptoms of dementia such as depression (Chu et al., 2014) and agitation (Raglio et al., 2010; Lin et al., 2011; Vink et al., 2013; Tsoi et al., 2018). Group music-based treatments (as distinct from music therapy), such as music listening and making or moving to music, have also been shown to improve overall cognition (Van de Winckel et al., 2004; Särkämö et al., 2014; Cheung et al., 2018; Tang et al., 2018), in addition to specific cognitive functions, such as attention and various executive functions (Särkämö et al., 2014) verbal fluency and memory (Cheung et al., 2018). Interestingly, group music- based activities designed specifically for cognitive stimulation in older adults with and without cognitive decline have been shown to offer greater benefits for cognition (measured using the MMSE) and executive function (such as verbal fluency and attention tasks) than physical activity alone (Biasutti and Mangiacotti, 2018). As with music therapy, group music-based treatments can also reduce symptoms of apathy (Tang et al., 2018), agitation (Choi et al., 2009; Ho et al., 2018) and depression (Ashida, 2000). Additionally, individualized music treatments, such as the use of personalized playlists, have been used to encourage cessation of antipsychotic medication (Thomas et al., 2017). Overall, it is clear that music has a multitude of ways in which it interacts as a therapeutic context for people with dementia.

Many interventions for people with dementia contain some of the same therapeutic qualities as music. For example, cooking and art therapy are engaging and social, and can invite personal reflection. In some instances, these therapies have offered similar benefits to music-based therapies. In one study, a cooking intervention and music intervention (involving singing and instrument playing) resulted in similar short-term reduction of behavioral disorders in people with AD (Narme et al., 2014), but it was only the music intervention that continued to have this effect long term. Other forms of therapy do not include some of music’s additional qualities that may lead to its extra therapeutic benefits. For example, music allows us to synchronize our actions, which promotes social bonding. Music can also induce spontaneous movement, which may confer cognitive benefits (Verghese et al., 2003). In addition, music has the innate ability to move us emotionally in the same manner as other stimuli that affect the hedonic centers in the brain, such as food and drugs (Blood and Zatorre, 2001). It is this combination of a number of capacities that makes music an ‘all in one’ therapeutic approach. Whilst this is one of music’s strengths, it also makes it a complex treatment tool to understand experimentally.

Experimental and review studies are contributing significant knowledge to devising successful music programs to improve dementia related symptoms. The majority of this research, however, has not identified the various capacities by which music can confer beneficial therapeutic effects. Moreover, there is no overarching theoretical model of the therapeutic value of music. To address this issue, Thompson and Schlaug (2015) proposed seven capacities of music that explain why it may be an ideal treatment tool for neurological disorders such as dementia. These seven capacities extend the framework outlined by MacDonald et al. (2012) who described ten qualities of music that may account for the links between music, health and wellbeing. As described in detail below, the seven capacities are that music is engaging, emotional, physical, personal, social, persuasive and permits synchronization. Each capacity represents a class of active ingredients of music-based treatments, and can be further analyzed into more concrete ingredients of effective interventions. Understanding these capacities, and associated active ingredients, should lead to more effective music interventions for people with dementia.

### The Seven Capacities of Music

Music is *engaging.* Music activates multiple systems in the brain simultaneously, including frontal, parietal, temporal, and cerebellar regions to deeper subcortical structures (e.g., Blood and Zatorre, 2001; Zatorre and Salimpoor, 2013). By engaging multiple processes, it places the brain in an ‘enriched’ and challenging setting, triggering neuroplasticity. In addition, by casting a ‘wide net’ of engagement, this offers multiple opportunities for addressing deficits. In particular, music can facilitate the encoding of verbal material by enhancing neural coherence during new learning (Peterson and Thaut, 2007). Whilst healthy individuals may not need to rely on music-enhanced encoding because they have intact cortical structures for memory, the mnemonic benefits provided by music may be necessary for individuals with AD (Kilgour et al., 2000). In effect, music provides a comprehensive, neurological scaffold for memory. Music also captures our attention such that we are likely to pursue the therapy in an undistracted manner, thereby reaping maximum benefits.

Music is *emotional*. One of the most significant purposes of music is to convey *emotional* meaning. Some brain regions involved in emotion processing, namely the medial frontal areas are relatively spared from degeneration in AD (Jacobsen et al., 2015). The ability of music to heighten emotions can be utilized to reduce apathy (loss of interest and a lack of or blunted emotional responses) in people with moderate to severe AD (Massaia et al., 2018; Tang et al., 2018). Receptive music-based treatments have been shown to significantly improve apathy (Massaia et al., 2018; Tang et al., 2018) and increase smiling behaviors compared to a control intervention of standard care (Raglio et al., 2008). Music also plays an important role in re-gaining access to emotions and memories, particularly in people with AD (e.g., El Haj et al., 2012; Baird et al., 2018). Interestingly, episodic memories evoked by music in people with AD tend to contain more emotional content and are more positively valanced, than episodic memories evoked in silence (El Haj et al., 2012; Cuddy et al., 2017), implying that the effects of music can benefit people with AD not only by eliciting memories, but also by inducing a positive state of mind.

Music is inherently a very *physical* stimulus. It is hard to separate the experiences of music and movement, and when we hear certain types of music we get a strong urge to move our body to the music. Engaging in physical exercise has been known to delay the onset of dementia (for review, see Laurin et al., 2001; Larson et al., 2006; Carvalho et al., 2014). Furthermore, in a longitudinal review over 5 years, engagement in leisure activities, such as dancing, reduced the risk of dementia (Verghese et al., 2003). Interventions that have paired music and exercise in people with dementia have reported a decrease in depression, as well as improvements in specific cognitive functions such as verbal fluency and memory (e.g., Cheung et al., 2018). Exercise and its associated benefits for memory may be accompanied by an increase in the production of brain derived neurotrophic factor which mediates neurogenesis (Erickson et al., 2011). Pairing music and movement therefore encourages exercise and subsequently benefits cognition, mood, and behavior.

Music affords *synchronization*. We have an instinctive ability to *synchronize* our body’s movements, and speech, to music. Simply moving in time with one another to music has many positive therapeutic benefits. For example, in synchronous drumming there is a release of endorphins and neurochemicals that are responsible for feelings of social bonding, empathy, and trust (Tarr et al., 2014). The tendency to move in time to music may assist in learning new movement sequences in people with AD (Moussard et al., 2014). Music treatments that have emphasized synchronizing the playing of musical instruments have resulted in improvement in cognitive functions such as verbal fluency, supported by neuroimaging results which demonstrate an increase in the level of cerebral blood flow to the prefrontal cortex (Shimizu et al., 2017).

Music is *personal* through its ability to reinforce our sense of self as it is commonly linked with our identity. Music that is heard repeatedly during significant or pivotal times in our personal development eventually seems to signify that time of life. There is increasing interest in using personalized playlists as a therapeutic tool for people with dementia (Garrido et al., 2017). Levels of agitation have been reported to decrease after listening to personally preferred music compared to relaxing classical music (Gerdner, 2000). Preferred music listening interventions can also reduce anxiety levels in people with dementia compared to standard care with no music (Sung and Chang, 2005; Sung et al., 2010). Familiar music can also be used to help people with dementia become more oriented within a new environment or maximize their sense of familiarity in a current one (Son et al., 2002).

Music is *social.* Isolation is one of the most significant challenges associated with dementia, owing to the decline in behavioral and cognitive functions. Music acts as a catalyst for bringing people together and also enhances group experiences. The *social* nature of music may be beneficial in boosting the healing process via cohesion, collective enjoyment and a sense of support for one another. Improvements have been demonstrated in cognitive functions (e.g., attention), behavior, mood, and wellbeing after participating in group singing and music activities (Sakamoto et al., 2013; Narme et al., 2014; Särkämö et al., 2014). Importantly, many people with dementia also indicate that group singing helped them to accept and cope with their condition (Osman et al., 2016).

Music is *persuasive*, and belief in a treatment is crucial for participation, motivation, and recovery. The positive belief in a treatment may make participating in therapy more likely (Rosenstock, 1974). In other words, merely believing that a treatment will lead to positive outcomes can amplify the therapeutic benefits. Music has the capacity to persuade or influence us and has been used historically as a tool to reinforce, change or inspire beliefs. For example, messages in advertisements or political movements are highlighted and enriched by music. It is persuasive also in the sense that the sheer enjoyment it stimulates leads to an optimistic outlook, which is beneficial in a therapeutic setting.

We have taken these seven capacities of music and developed the TMCM (Figure 1) (Brancatisano and Thompson, in press). The model begins by identifying contexts in which music can be experienced in a therapeutic way, previously identified by MacDonald et al. (2012). These contexts are broken down into the seven capacities that form the core of the model. A number of biological and psychological processes are then listed that may underlie the link between the seven individual capacities of music and their beneficial outcomes. Finally, arising from the seven capacities through the underlying mechanisms are multiple potential benefits, including cognitive, psychosocial, motor, and behavioral benefits. As it stands, most music-based treatments or music therapy practices incorporate one or more of these attributes, but not all. These capacities have not yet been combined to form an intervention in a systematic way, which may in turn maximize the effect of a music-based intervention.

We devised the MMM program based on the TMCM. The MMM program was then evaluated in people with dementia to (a) determine the potential benefits that including all seven capacities of music would have on the participants’ cognition, mood, identity and motor function, (b) examine the impact that certain individual or a combination of capacities would have on the participants’ cognition, mood, identity and motor function, and (c) explore the relationship between factors which may affect session attendance and cognitive performance.

## Materials and Methods

### Ethical Approval

Ethical approval was granted by the Macquarie University Human Sciences Ethics committee and the residential aged care facility. Verbal consent to approach the participants was first sought from their significant other (family member or partner). Written and informed consent was obtained from all participants, who underwent an initial visit to explain the study and gauge their interest. If participants were interested, a second visit was organized for verbal and written consent from the participant and their significant other. Continuing consent was monitored each week with each participant being re-informed of the nature of the program and asked if they were happy to participate. If the participant declined, they were invited again to take part the following week. If they declined 2 weeks in a row, they were excluded from the study.

### Participants

We recruited participants from a local residential aged care facility who had either a clinical diagnosis of any type of dementia (mild to moderate) or indication of cognitive impairment (e.g., mild cognitive impairment, ‘memory impairment’) as specified in their medical records. Evidence of probable dementia was confirmed in all participants by cognitive assessment results using Addenbrooke’s Cognitive Examination III (ACE-III total score at or below 82). Only one participant scored above 82 (a score of 84) which was indicative of possible dementia. Inclusion criteria were fluent English language skills, no severe psychiatric disorder (e.g., schizophrenia) and no hearing or language impairment that would prevent communication or ability to hear music. We approached the residential aged care facility manager who provided a list of 44 names of individuals who matched these criteria. The potential participants’ family members were then contacted, fourteen of whom declined to participate. Thirty participants were then visited individually at the residential facility to determine their suitability and interest in the program. Ten participants refused to participate leaving a final sample of 20. These 20 participants then completed baseline assessments.

The 20 participants were divided into two groups for logistical reasons. Group 1 completed the whole MMM program first and Group 2 completed the program second (immediately after Group 1), with Group 2 acting as a ‘waitlist control.’ The demographic and clinical characteristics of the participants are presented in Table 1. The clinical diagnoses were obtained from the medical records at the residential facility. In 10 out of 20 participant no formal diagnosis of dementia noted in the medical records, but the ACE-III scores revealed that all participants met the cut off for probable dementia. The two groups were well matched in that they included various dementia types and one participant in each group had a diagnosis of Parkinson’s disease and associated dementia (see Table 1).

**TABLE 1:** Demographic and clinical characteristics of the two groups of participants (*n* = 20).

	**Group 1 (*n* = 10)**	**Group 2 (*n* = 10)**
Age (years)	84.4 (7.1)	82.20 (8.0)
Gender (M/F)	3/7	1/9
Education (years)	16.0 (0.9)	15.11 (1.0)
Time at residential facility (months)	27.2 (20.2)	18.20 (18.5)
Musical background (yes/no)	4/6	4/6
Clinical diagnoses (number of participants)		
Alzheimer’s dementia	3	2
Vascular dementia	2	1
Mild cognitive impairment	0	2
Memory disturbance	4	2
Other	1	3

*Data presented as mean (standard deviation) unless otherwise stated. Memory disturbance specified as ‘amnesia,’ ‘memory changes/issues,’ ‘short term memory loss’ on medical records. Other specified as mood disorders (*n* = 1), Parkinson’s disease (*n* = 2), depression (*n* = 1).*

### Conditions

#### The MMM Program

The MMM program incorporates music-based activities that highlight all seven capacities of music. The capacities represent distinct *qualities* of music and the outcomes are the *benefits* that arise as a consequence of tapping into these capacities to target relevant areas of decline, such as cognitive, behavioral, motor, and identity, as outlined in the TMCM (see Figure 1). The MMM program encompasses activities that possess a specific set of ‘active ingredients’ to enrich the environment and maximize the activation of the seven capacities outlined in the TMCM. We suggest that there are five active ingredients that apply to various activities in the MMM program:

(a) Familiarity of the music (novel or familiar)
(b) Complexity of music activity (number of functions engaged in an activity)
(c) Intensity of music activity (quantity of each task)
(d) Difficulty of music activity (easy or hard)
(e) Empathic focus (degree to which an activity engages empathy)

Through the activities, participants are encouraged to play simple instruments (such as bucket drums and egg shakers), sing, move and interact with one another.

##### Sessions

We devised seven weekly sessions broken into four blocks and each block contained activities that focussed on two or three related capacities of music (a summary of the activities can be seen in Table 2). The four blocks were designed to determine the relative contribution of each individual or combination of comparable capacities on specific outcomes, for example, the contribution of the personal and emotional capacities of music (sessions 3 and 4) related to the outcome of ‘mood’ and ‘identity’ (Table 2).

**TABLE 2:** MMM sessions and their targeted capacities, active ingredients, and outcomes.

	**Sessions 1 and 2**	**Sessions 3 and 4**	**Sessions 5 and 6**	**Session 7**
Capacities	*Engaging* and *persuasive*	*Emotional* and *personal*	*Social*, *physical* and *synchronization*	*All attributes*
Active ingredients	Familiarity, Novelty, Complexity, Simplicity, Empathic focus	Familiarity, Empathic focus	Familiarity, Complexity, Intensity, Novelty	All ingredients
Example activities	*Novelty, Simplicity and Complexity* (i) Introducing each musical instrument with songs (egg shakers, drums) (ii) Learning names and personal description to melodic and rhythmic framework *Familiarity* (i) Game: Guess that advertisement’s song (and singing along) *Empathic focus* (i) Game: Which advert jingle is more convincing? (discussing how music makes adverts convincing) (ii) Game: Where does this song belong? (guessing the context of the song and discussing why it fits there)	*Familiarity* (i) Game: Who sang that song? (guessing the name of artist and song) (ii) Finish the next line of the song lyrics (singing along to favorite songs and guessing the missing lyrics) *Familiarity and Empathic focus* (i) Reminiscence with music (play each individual’s favorite sons and discuss memories evoked) (ii) Game: Guess which movie the soundtrack belongs to? (iii) Music and our emotions (playing to express emotions and how you are feeling)	*Familiarity and simplicity* (i) Warm up movement exercises; start with slow songs (e.g., “My Bonnie”) and progress to faster (e.g., “In the Mood”) *Familiarity, Complexity, Intensity* Musical instrument playing (moving and making music in synchrony, turning to face partners and exchanging instruments and making eye contact). *Novelty* Game: Match the dance song to the photo (discuss why the music fits with each song) **	*All ingredients* Combine activities from other sessions (e.g., reminiscence, singing, playing along and engaging socially).
****Outcomes	*Cognitive* (memory and attention)	*Psychosocial* (mood and identity)	*Motor* (verbal and movement fluency)	All

Block one (sessions 1 and 2), focussed on the *engaging* and *persuasive* attributes which may benefit attention and memory functions. The active ingredients in these sessions include familiarity, novelty, complexity, simplicity, and empathic focus. For example, in the first session the activity is relatively simple, as the participants attempt to complete verbal tasks to novel musical structures (e.g., how melody and rhythm can be used to help to remember verbal information, such as names or short phrases). Participants are invited to listen to how messages in familiar advertisements are highlighted and enriched by music, making them extremely memorable.

Block two (sessions 3 and 4) focussed on the *emotional* and *personal* attributes. The active ingredients in these sessions are familiarity and empathetic focus, which are designed to facilitate mood and identity. Familiarity is emphasized by including the participants’ personally selected favorite songs as well as songs from their reminiscence bump period. The aim of this block is to encourage reminiscence and discussion about personal memories and to stimulate emotions through playing and listening to music, embodying empathy.

Block three (sessions 5 and 6) emphasized the *social*, *physical* and *synchronous* attributes. The active ingredients in this session are familiarity, simplicity, complexity, and intensity to stimulate positive change in both motor and verbal fluency. For these sessions, we used familiar music that had a strong beat and paired it with body movements to promote physical activity. Participants were encouraged to play their instruments in synchrony with each other and to engage socially whilst doing so (such as turning to the person beside them). Rhythmical phrases played in time to the music started off simply, to allow participants to gain a sense of mastery, and became more complex as the session progressed.

Block four (session 7) drew upon all the capacities and involved live music, encompassing all active ingredients. As it was the last session, an emphasis was placed on intensity and familiarity. The active ingredients in this session were ‘intensive’ and ‘familiar,’ as it is the last session. Musicians from the local Conservatorium of Music played the residents favorite songs. Live music can be especially engaging for participants and playing preferred and familiar songs may help to bring back personal memories and provide a rich emotional experience. During this session, participants were encouraged to respond to the music with physical movements and singing along, allowing them to synchronize their movements and voice with the music and with other participants, ensuring that they would have a powerfully social experience.

##### Music choice

Before starting the program, the researchers asked the participants for at least one or two favorite songs, artists or genres of music. This was primarily for the reminiscence sessions in Block 2. All other music used in the sessions was from the participants reminiscence bump period (aged 10–30 years). This would ensure the participants would be highly familiar with the songs and the personal nature of the program would be maximized. A list of songs used in the program can be found in Appendix.

##### Musical instruments

Each participant had a bucket drum, two drum sticks and an egg shaker set up in front of them at the start of each session. The researchers ensured they were easily accessible (for example, if the participants had trouble reaching the bucket drum two or more buckets were stacked on top of each other).

##### Program set-up

The duration of each session was approximately 45 min. This allowed approximately 10 min for each activity (see Table 2). The sessions took place in a medium sized room at the residential aged care facility. Power-point slides were devised to both prompt the researchers and show pictures and videos to the residents (mainly for sessions 1–4). Participant chairs were set up in a semi-circle to face the projector screen where the power-point slides were displayed. The intervention was conducted by two facilitators. Facilitator one (F1, JC) had experience with prior group music programs in schools and elderly care, and facilitated all sessions. The second facilitator (F2, OB), assisted by encouraging discussion and ensuring all participants had the relevant musical instruments. A third facilitator (F3, AB) was present for Group 1 and had a similar role to F2. One facilitator would typically sit at the front by the projector screen and the other approximately half way around the circle to facilitate a social environment.

Approximately half of the participants required wheelchairs for transportation to and from the venue and stayed in their wheelchair during the sessions. These participants demonstrated a minimal to medium range of motion and were able to move upper and lower limbs voluntarily.

##### Booster sessions

On a separate day after each group session, F1 visited each participant individually for 10–15 min as part of the ‘Booster Sessions.’ The booster sessions were designed as a way to increase dosage and allow the participants to reflect on their experience of the previous session. F1 would first ask the participants a series of open-ended questions pertaining to whether they remembered the previous MMM session, and if so, did they enjoy it and specifically what they enjoyed about it. F1 then engaged the participant in completing two activities that were covered in the previous session as a “refresher” for the participant.

#### Standard Care

Group 2 served as a waitlist group for 7 weeks and continued their standard care and activities until their follow-up assessments. Activities that were available to them by the residential aged care facility on a weekly basis were group discussions, miniature bowling, physical exercise, ‘name that tune’ and craft. Nine out of the 10 participants answered a series of yes/no questions to determine their level of activity during this period of time; 2/9 participants said they participated in physical exercise; 5/9 participants said they engaged in social activities; 3/9 participates said they sang regularly; 6/9 participants said they listened to music regularly and 0/9 participants said they played a musical instrument currently.

### Design

**FIGURE 2:**
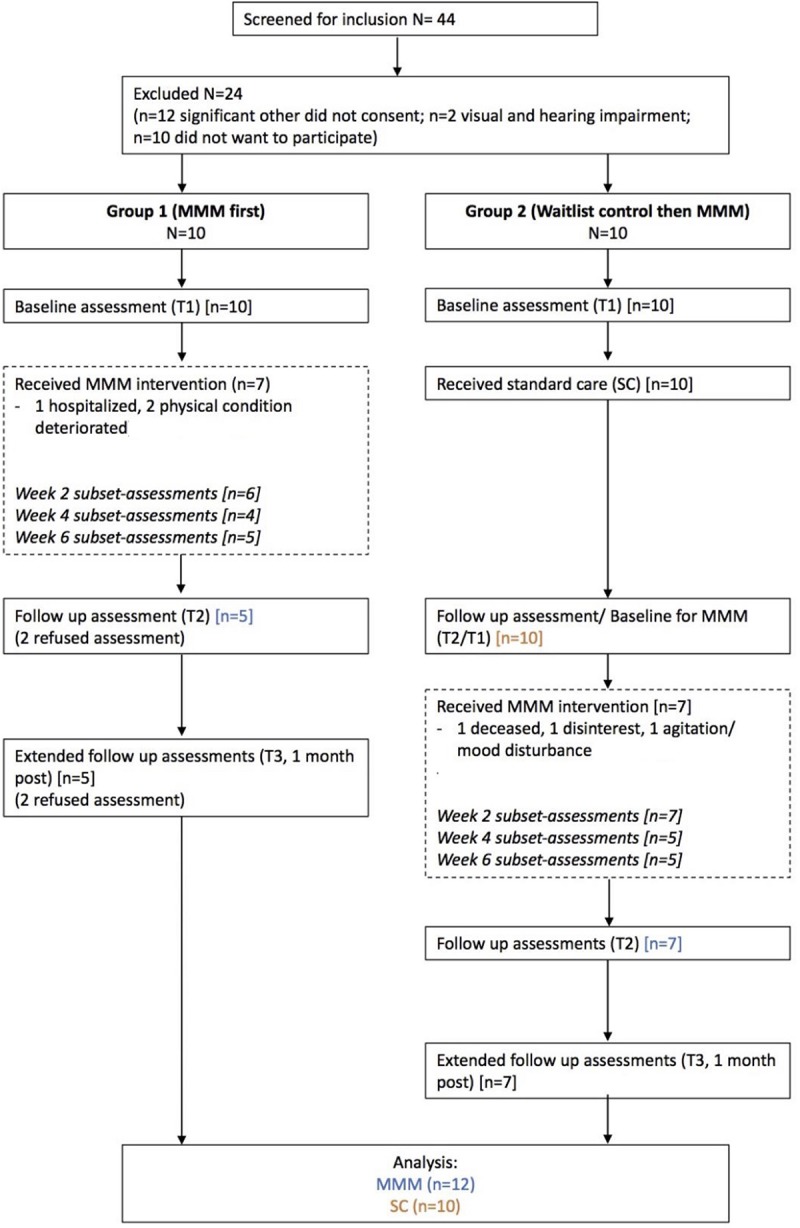
Flow chart of the study design.

The study design is illustrated in Figure 2. Two groups of participants completed the MMM program (Group 1 and Group 2). Group 1 completed the MMM program first, whilst recruitment of Group 2 took place. Group 2 then served as a waitlist control, with a period of no intervention during which they received standard care only (henceforth referred to as the SC group). Group 2 then completed the MMM program immediately after Group 1. All participants who completed the MMM program are henceforth referred to as the ‘MMM group.’ Assessments of global cognition, mood, identity, verbal and motor fluency were taken at baseline (1 week prior to MMM program/SC start, T1) and at a follow-up immediately after the program/SC (T2). For the MMM group only, assessments were also taken at an extended follow-up 1 month after participation in the program (T3).

A subset of the measures (brief assessments for a total duration of 5 min) were taken at the end of the last session of the three MMM blocks (in week 2, 4, and 6). This was designed to assess the distinct effect that each of the seven attributes might have on specific domains of functioning. Baseline assessments of Group 1 were performed by authors 1 and 3 (F2 and F3; OB and AB, respectively). Baseline assessments of Group 2 were performed by an independent researcher, blind to participant group membership, who also conducted all other follow-up assessments for both groups (excluding Group 2 follow-up SC assessment, due to logistic restrictions which were done by F2 who was blinded to the original baseline assessment scores).

### Measures

#### Cognitive Function: Addenbrooke’s Cognitive Examination (ACE-III) Australian Version III

The ACE-III is a cognitive measure used in screening for dementia and developed as a “theoretically motivated extension of the MMSE” (Mathuranath et al., 2000; Mioshi et al., 2006). Cut-offs at 88/100 (sensitivity = 94%, specificity = 89%) and 82/100 (sensitivity = 84%, specificity = 100%) for the suspicion of dementia have been defined. The ACE-III has very good reliability (alpha coefficient = 0.8).

The ACE-III tests five subdomains of cognitive skills: attention (/18), memory (/26), verbal fluency (/14), language (/26), and visuospatial skills (/16) with a total score out of 100. The subdomain of attention tests the participants’ ability to: recall the date, the current season and location, repeat back immediately three simple words and serial subtraction. The memory items first test the participants’ ability to: recall the three simple words, then asks them to verbalize them, memorize and recall a fictional name and address, and remember several well-known historical facts. Fluency tests the ability to: list as many animals they can in 1 min, followed by as many words they can beginning with the letter ‘P’ in 1 min. The language subdomain requires the participant to: complete a series of physical tasks that are verbally directed by the researcher using a pencil and piece of paper (e.g., “pick up the pencil but not the paper”), write two complete sentences, repeat four complex words and two short proverbs, name 12 simple drawings of objects and animals, answer four semantic questions relating to the drawings, and read aloud five words that are easily mispronounced. Lastly, the visuospatial abilities subdomain tests participants’ ability to: copy two figures, draw a clock face and the hands set at a particular time, count multiple dots in a square and recognize four partially obscured letters. Questions in the attention, memory and verbal fluency domains were also taken at the end of week 2, and verbal fluency domain at the end of week 6.

#### Mood: Geriatric Depression Scale Short-Form (GDS-SF)

The Geriatric Depression Scale Short-Form (GDS-SF) has been used to screen for depression in the elderly (Sheikh and Yesavage, 1986). It was initially developed as a 30-item tool but the short form (15-item) was established for use in time constraints. The GDS-SF has been reported to be sensitive to depression in people with dementia. Scores greater than 5 points suggest depression and scores greater than 10 are almost always depression. It has good sensitivity (92%), specificity (89%), and high correlation (*r* = 0.84, *p* < 0.001).

#### Self-Identity: ‘I Am/I Was’ Task

In the original form of this task (Rathbone et al., 2008), participants are asked to list 10 ‘I am’ statements that strongly define their identity (e.g., “I am a grandfather,” “I am shy”). They are then asked to select the three most relevant statements and recall a personal memory that is linked to each one. For this study, the task was modified to involve the participant listing as many ‘I am’ and ‘I was’ statements as they could in 1 min per category (e.g., “I was a dancer,” “I was a nurse”) in order to measure the participant’s change in identity over time. We also omitted the task of recalling memories associated with the statements and adapted the task for people with dementia by asking them to list the statements verbally rather than in written form, similar to that of Gridley et al. (2016). This task was also repeated at the end of week 4.

#### Autobiographical Memory Fluency: Autobiographical Fluency Task (AFL)

In the original version of this task (Dritschel et al., 1992), participants are asked to recall list personal events and names of friends/acquaintances from different lifetime periods: ages 5–11 years, 11–17 years, 5 years post high school and currently. They are given 90 s for each stage of life and each category (events and names). For this study, we omitted the events category and asked participants to list names only. This task was also repeated at the end of week 4.

#### Motor Fluency: Nine-Hole Pegboard Task

This task was initially introduced by Kellor et al. (1971) and is used to measure finger dexterity in neurological disorders, particularly stroke and Parkinson’s disease. Participants were timed on how long it takes to insert all nine pegs one by one into the holes and remove them one by one. Measurements were taken for both the dominant (self-reported preferred writing hand) and non-dominant hands. This task was also repeated at the end of week 6.

### Data Analysis

Data analysis was completed using SPSS. Due to the cross-over structure of the study, the small sample size and several variables violating the assumptions of normality, we analyzed data using the Wilcoxon Signed Rank test within the SC and MMM groups. We compared the outcome measures (cognition, mood, self-identity, autobiographical memory fluency and motor fluency) within the MMM and SC groups at T1 versus T2, and T2 versus T3 (MMM group only) to determine the effects of the MMM program overall.

We also sought to determine the distinct contribution that each of the seven capacities had on a subset of outcome measures. To do this, we conducted pairwise comparisons of assessments taken at baseline and ‘test’ phase (either week, 2, 4, or 6 of the MMM program). At the end of week 2 (following sessions focussing on engaging and persuasive capacities) assessments consisted of subsets of cognition (attention, memory and verbal fluency). At the end of week 4 (following sessions on the personal and emotional capacities), assessments consisted of tasks assessing autobiographical fluency (AFL) and identity (‘I am/I was’ task). Finally, at the end of week 6 (following sessions on the social, physical, and synchronous capacities) assessments consisted of motor fluency (peg hole task) and verbal fluency tasks.

The answers provided by the participants to the open-ended questions during the booster sessions were categorized into each of the seven capacities of music. This was completed by F2, according to the key words the participant used to describe what they enjoyed. For example, if participants said that they “enjoyed moving to the music” this was categorized into the *physical* capacity. If participants stated that they “enjoyed talking with one another” or “being a part of something” this was categorized into the *social* capacity. The categories of the seven capacities of music were not mutually exclusive, as some responses included many key words describing several capacities. For example, some participants stated that they “enjoyed talking about old memories, with everyone and hearing their stories” this was classified as *personal* and *social.*

## Results

### Group Characteristics

There were no significant differences between the Group 1 and Group 2 in age, gender, level of education, or musical background (whether they had spent time learning an instrument or singing, see Table 1).

### Drop-Out Rates

Figure 2 shows the drop-out rates per week for the MMM group and the associated reasons. In Group 1, 7/10 participants completed the MMM program (three drop-outs due to hospitalization and deterioration of physical condition) and in Group 2, 7/10 participants completed the MMM program (three drop-outs due to death, disinterest and agitation/mood disturbance). Regarding post program assessment, in Group 1, five participants were included in the analysis (two refused final assessment). In Group 2, all 10 participants completed assessments pre/post the waitlist period of standard care, and all seven participants who finished the MMM program completed post assessments. Thus, analyses of cognition, mood, identity and fine motor skills at T1, T2, and T3 were conducted on 12 participants in the MMM group (five from Group 1 and seven from Group 2) and 10 participants in the SC group.

We compared the participants who dropped out of the MMM program (*n* = 6) with those who completed the intervention (*n* = 12) on several potential influencing factors such as age, duration of residing at facility and total ACE-III score (pre MMM intervention). There were no significant differences between those that dropped out and those who completed the intervention on any of these measures.

### Cognitive Function

Table 3 shows results for total scores and subdomain scores of ACE-III for participants in the SC and MMM groups. We examined both the total ACE-III scores and the ACE-III subdomain scores for the period of standard care (*n* = 10) and for the MMM program (*n* = 12), comparing the scores at T1 versus T2, and T1 versus T3 (for MMM group only).

**TABLE 3:** Outcome measures, mean (SD), for standard care and MMM groups at T1 (baseline), T2 (follow-up), and T3 (extended follow-up, MMM only).

**Standard care (*n* = 10)**	**MMM (*n* = 12)**						
	**T1**	**T2**	**T1**	**T2**	**T3**	
Total ACE-III score (/100)		59.40 (24.87)	55.20 (22.56)^*^	57.00 (16.64)	60.58 (18.85)	60.50 (18.54)
Attention (/18)	12.00 (4.83)	10.70 (4.42)	10.67 (3.20)	12.92 (3.20)^∗∗^	11.42 (3.89)	
Memory (/26)	14.20 (6.23)	13.40 (8.45)	12.08 (6.23)	12.75 (5.99)	14.08 (5.42)	
Fluency (/14)	5.80 (3.82)	3.90 (3.14)	4.33 (2.74)	5.58 (2.39)^*^	5.0 (2.92)	
Language (/26)	18.70 (8.37)	19.40 (7.04)	20.58 (4.83)	20.42 (5.93)	20.5 (5.93)	
Visuospatial (/16)	8.70 (4.44)	7.80 (3.88)	9.42 (4.38)	8.92 (4.29)	9.5 (3.87)	
GDS-SF (/11)		5.33 (2.91)	5.89 (3.10)	3.83 (2.29)	4.17 (2.59)	3.33 (2.10)
Identity statements						
‘I was’		7.10 (3.48)	5.40 (3.06)	6.33(2.81)	6.67 (3.82)	5.92 (3.34)
‘I am’		5.20 (1.75)	3.70 (2.83)	3.67 (2.71)	4.75 (1.71)	4.58 (3.31)
Autobiographical fluency score (total)		12.00 (10.99)	10.50 (10.28)	9.92 (9.95)	10.75 (9.84)	10.58 (12.97)
Peg task (seconds)						
Dominant hand	53.58 (10.67)	69.89 (17.48)	58.3 (15.91)	56.84 (23.12)	49.93 (5.78)	
Non-dominant hand	55.34 (16.03)	62.64 (12.43)	61.31 (10.09)	61.67 (21.61)	54.66 (10.98)	

Data presented as mean (standard deviation) unless otherwise stated. ^*^p < 0.056 (T2 vs. T1), *^∗∗^p* < 0.007 (T2 vs. T1).

**FIGURE 3:**
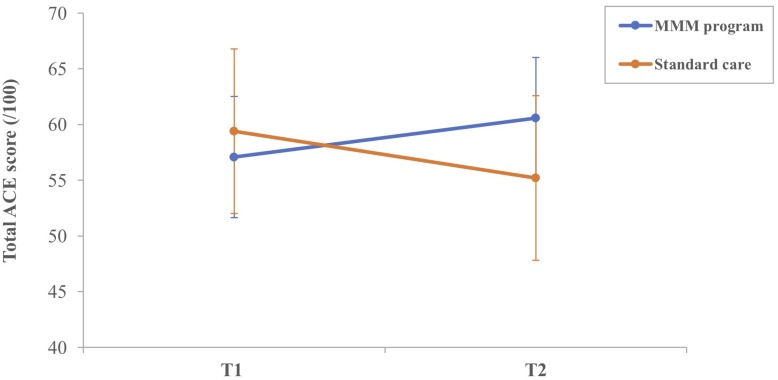
Mean total ACE-III score for participants in the MMM (*n* = 12) and standard care (*n* = 10) groups at T1 (time 1, baseline) and T2 (time 2, follow-up) (error bars = mean ± standard error of the mean).

On average, there was a decrease in the mean total ACE-III score for participants in the SC group from T1 (59.40) to T2 (55.20). The majority of participants (8/10) in the SC group showed a decline in total ACE-III score, eliciting a marginally significant median decrease, *z* = −1.89, *p* = 0.059. For participants in the MMM group, the mean total ACE-III scores improved between T1 (57.00) and T2 (60.58). Overall, 8/12 participants had an increase in total ACE-III scores after participating in the MMM intervention, and the remaining four participants had a decrease. Statistical analysis showed a marginally significant median increase in total ACE-III scores, *z* = −1.93, *p* = 0.054 (Figure 3).

The MMM group showed an increase in mean total ACE-III score from T1 (57.00) to T3 (60.50), 1 month after the MMM program. Again, 8/12 participants in the MMM group had an increase in total ACE-III scores 1 month after the MMM program, and the remaining four participants had a decrease, but this median difference did not reach statistical significance.

**FIGURE 4:**
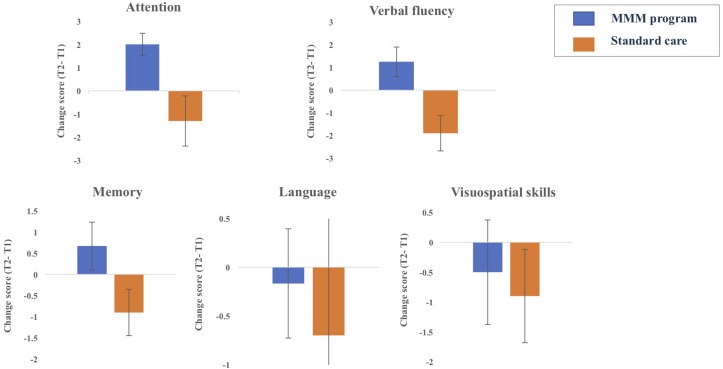
Changes in the five ACE-III subdomain scores from T1 (time 1, baseline) to T2 (time 2, follow up) for participants in MMM (*n* = 12) and standard care groups (*n* = 10) (error bars = mean ± standard error of the mean).

Statistical analysis on the ACE-III subdomain scores revealed there was no significant change in attention, memory, fluency, language or visuospatial ability scores from T1 to T2 in the SC group. In contrast, the MMM group showed an increase in mean ‘attention’ scores from T1 (10.67) to T2 (12.92). Nine participants showed an improvement in attention scores, three showed stable scores and no participant showed decreased attention scores, producing a statistically significant median increase in the subset of attention, *z* = −2.68, *p* = 0.007. There was also a slight increase in mean ‘verbal fluency’ scores from T1 (4.33) to T2 (5.58). Seven participants showed an improvement in verbal fluency scores, three showed stable scores and two participants showed decreased scores, producing a marginally significant median increase in the subset of verbal fluency, *z* = −1.91, *p* = 0.056 (Figure 4).

Comparisons of the ACE-III subdomain scores between baseline (T1) and the extended follow-up (T3) for participants in the MMM program revealed there was no significant differences in the subdomain ACE-III scores of attention, verbal fluency, memory, visuospatial skills or language between the time intervals (T1 and T3). When observing individual total ACE-III scores from T1 to T3 in the MMM group, we found that the total ACE-III score increased for eight out of the 12 participants after the extended follow up, whereas four participants exhibited a decrease in these scores.

Whilst the design of our study did not permit direct between- group statistical comparisons, Figure 4 illustrates the changes in the five ACE-III subdomains over the 8-week period for participants in the SC and MMM groups. We can see that whilst participants in the SC group decreased slightly in attention, memory and verbal fluency scores, participants in the MMM group showed slight increases in these three subdomain scores. Both groups had a negligible decrease in language and visuospatial subdomains scores over the 8 weeks, with the SC group showing a slightly greater decline.

### Mood

The mean GDS-SF scores of participants in both the SC and MMM groups remained stable between T1 and T2 and, T1 and T3 for the MMM group. In other words, there was no difference in reported depression symptoms before and after the period of intervention or standard care (see Table 3).

### Self-Identity

All 20 participants were able to generate ‘I am/I was’ statements at T1. However, one participant, who had minimal expressive language at T1, was unable to generate any statements at T2 after the period of standard care. This participant later withdrew from the MMM program at week 2. The mean total number of ‘I am’ and ‘I was’ statements were analyzed separately. There were no significant differences in the mean number of ‘I am/I was’ statements between T1 and T2 in the SC or MMM groups, and T2 and T3 in the MMM group (see Table 3).

### Autobiographical Memory Fluency

For analysis purposes, the total number of names listed in each lifetime period was collapsed to create a total AFL score. There was no difference in the total number of names produced by participants in the SC and MMM groups at T1 compared with T2, and T2 compared with T3 in the MMM group (see Table 3).

### Motor Fluency

Within the SC and MMM groups we compared the time taken (in seconds) for participants to complete the pegboard task with their dominant and non-dominant hand at T1 versus T2. In the SC group, there was a trend for the time taken to complete the motor task to increase for both the dominant and non-dominant hands (from 53.58 s to 69.89 s, and from 55.34 s to 62.64 s, respectively), but these differences did not reach statistical significance. In the MMM group, the time taken for participants to complete the task with the dominant and non-dominant hands remained stable from T1 to T2. For participants in the MMM group, there was a trend for the time taken to complete the task with the dominant hand to decrease, from 56.84 s (T2) to 49.93 s (T3), but this was not statistically significant (Table 3).

### Assessment of Individual Seven Capacities

To determine the distinct contribution that each of the seven capacities had on the subset measures we conducted pairwise comparisons between assessments taken at baseline and test phase (either week, 2, 4, or 6). Table 4 displays the assessments conducted at week 2, 4, and 6. Results showed that there were no significant differences between baseline and test phase on any of the subset of measures at week 2, 4, or 6.

**TABLE 4:** Subset measures, mean (SD), for participants who completed measures at the relevant sessions (baseline and test phase) during the MMM program.

**Measures**	**Baseline**			**Test phase**		
Engaging and persuasive	Attention (/8)	Memory- word list (/3)	Verbal fluency- letter (/7)	Attention (/8)	Memory-word list (/3)	Verbal fluency- letter (/7)
Week 2 (*n* = 12)	3.58 (1.83)	0.92 (1.16)	2.83 (0.39)	3.50 (1.38)	1.17 (1.11)	2.67 (1.50)
Emotional and personal	AFL total	‘I was’	‘I am’	AFL total	‘I was’	‘I am’
Week 4 (*n* = 11)	6.0 (5.06)	5.27 (2.57)	3.73 (2.24)	5.54 (4.76)	5.18 (3.06)	4.0 (2.0)
Physical, synchronous and social	Peg task (dominant)	Peg task (non-dominant)	Verbal fluency-total (/14)	Peg task (dominant)	Peg task (non-dominant)	Verbal fluency-total (/14)
Week 6 (*n* = 10)	55.30 (23.45)	56.64 (15.07)	3.0 (1.43)	47.25 (10.20)	43.25 (12.51)	4.0 (1.45)

*AFL, autobiographical fluency task; dominant, dominant hand; non-dominant, non-dominant hand.*

### Exploratory Analyses

#### Prediction of Change in Cognition

We wanted to determine whether certain variables (age, years of education, time in residency and, number of sessions attended for MMM group only) predicted the change in cognition from T1 to T2, as measured by the total ACE-III score. To do this, we calculated a ‘change score’ by subtracting the total ACE-III score at T1 from the total ACE-III score at T2. We then conducted a multiple regression analysis to determine if there was a relationship between these variables and the total ACE-III change score for participants in both MMM and SC groups.

**FIGURE 5:**
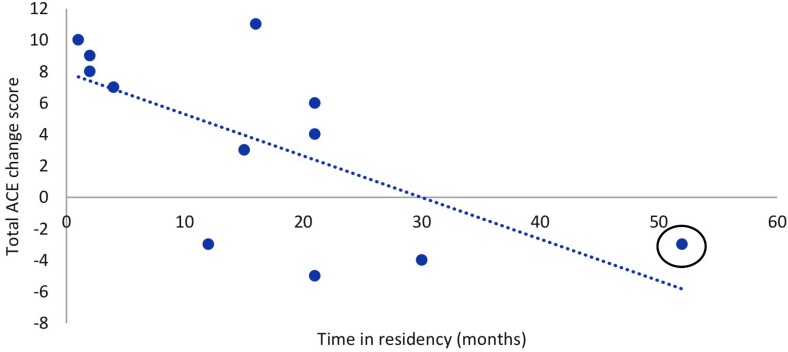
Relationship between time in residency and total ACE-III change score (T2–T1) after the MMM intervention (*n* = 12). Outlier circled.

In the MMM group, there was a statistically significant regression equation, *F*(3,8) = 4.78, *p* = 0.034, adj. *R*^2^ = 0.508. The only variable to predict change in total ACE-III score was the participants’ time in residency, *p* = 0.040 (Figure 5). In other words, the longer the participant had been a resident at the aged care facility the less improvement in overall cognition after the MMM program. Age, years of education, and number of sessions attended were not significant predictors. For participants in the SC group, the model was non-significant, demonstrating no relationship between any of the demographic variables (age, years of education, and time in residency) and the total ACE-III change score.

We then further examined the significant negative relationship between participants’ time in residency and the total ACE-III change score the MMM group. We removed the participant with the longest duration of residency (52 months), their being an outlier by more than 2 standard deviations (see Figure 5). With this outlier removed, the data did not violate the assumption of Normality determined by the Shapiro–Wilk statistic. We then reanalyzed the comparison of total ACE-III scores pre and post the MMM program. We found a statistically significant increase in total ACE-III scores from T1 (*M* = 56.27, *SD* = 17.25) to T2 (*M* = 60.45, *SD* = 19.76), *t*(10) = −2.4, *p* = 0.037, and from T1(56.27) to T3(*M* = 61.18, *SD* = 19.29), *t*(10) = −3.03, *p* = 0.013 for participants in the MMM group.

#### Prediction of Attendance of MMM Sessions

**FIGURE 6:**
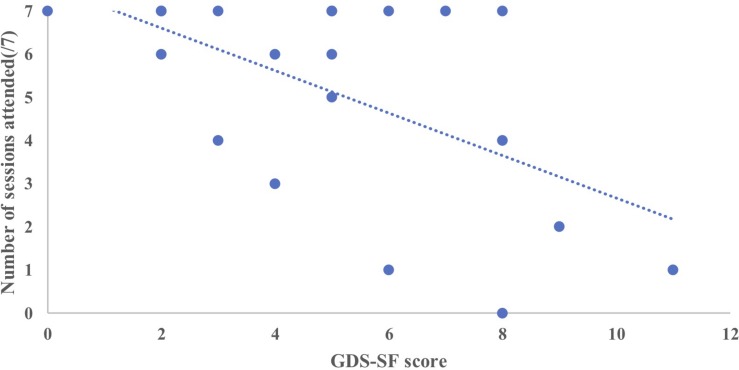
Relationship between the number of MMM sessions attended (maximum 7) and GDS-SF score prior to starting the intervention (*n* = 20).

To examine predictors of session attendance in the MMM program we conducted a multiple regression analysis with the variables of age, time in residency and GDS-SF score. The multiple regression model statistically significantly predicted the number of MMM program sessions attended *F*(2,17) = 4.79, *p* = 0.022, adj. *R*^2^ = 0.285. The GDS-SF score was the only variable to significantly predict MMM session attendance, *p* = 0.017. In other words, the higher the GDS-SF score (indicative of possible depression), the less MMM sessions attended (Figure 6).

We then compared the GDS-SF scores of the participants who dropped out of the MMM program with those who completed the program. Those who dropped out of the MMM program had a higher GDS-SF score prior to the MMM program (7.33), suggestive of mild depression, compared to those who remained in the program, who had no indication of depression prior to the MMM program (4.21), *t*(18) = 2.64, *p* = 0.017.

#### Subjective Responses From Booster Sessions

**FIGURE 7:**
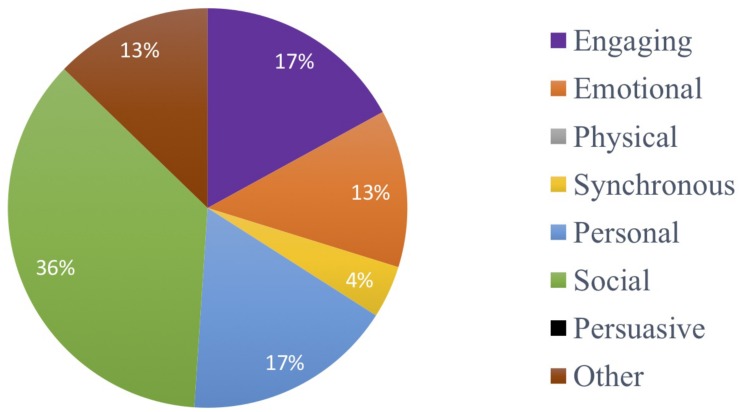
Distribution of the participants’ responses as to what aspects they enjoyed in the MMM program (social, 36%; engaging, 17%; personal, 17%; emotional, 13%; other, 13%; synchronous, 4%; physical, 0%; persuasive, 0%).

The weekly booster session visits were made by F1 to participants who attended the corresponding MMM session. The availability of participants varied on this day and was influenced by factors such as health, visitors and their activity schedule. Thus, 67.3% of participants on average each week were seen for booster sessions. Figure 7 depicts the distribution of the participants’ responses to the open-ended questions determining what aspects they enjoyed in the MMM program. The social capacity of music in the MMM program was the most valued by the participants, accounting for 36% of the responses. These responses included statements about enjoying the company of others in the group, making new friends, being in a group scenario, participating in discussions or ‘getting out of their room.’ The engaging and personal capacities were the second most common theme of responses each accounting for 17% of the responses. These included statements about enjoying the experience of learning new things and the sounds of the musical instruments. Responses pertaining to the personal capacity included enjoying reminiscing and listening to their old songs. References to the emotional capacity of music were present in 13% of responses and included statements of feeling “lifted,” “happy,” and “ready to party” after the sessions. Finally, 4% of responses included reference to the synchronous capacity of music and included statements such as doing two actions at one time. Other types of reflections accounted for 13% of all responses and included statements pertaining specifically to the MMM program, for example that it was “entertaining,” “something different’ and that the program was “delivered well.”

## Discussion

We conducted a pilot MMM program for people with mild to moderate dementia (of various types) and examined its effects on cognition, mood, identity and fine motor function. The MMM program is group-based, comprising of seven weekly sessions broken into four blocks which draw upon the seven capacities of music in the TMCM (Figure 1). The main finding was that compared with people receiving standard care who showed a slight decline in global cognition (ACE III total score) over time, those who participated in the MMM program showed a slight improvement in global cognition. In particular, significant improvements occurred in the subdomain scores of attention and verbal fluency. In contrast, we did not find any change in mood, sense of identity or motor function after MMM participation. To our knowledge, the MMM program is the first music program based on a theoretical model underpinning the beneficial capacities of music for neurological impairment.

Our primary finding was a marginal improvement of global cognition after the 7-week MMM program. This result is in keeping with previous studies which have shown that both music-based interventions and music therapy can improve or stabilize global cognition in people with dementia (Suzuki et al., 2004; Van de Winckel et al., 2004; Bruer et al., 2007; Chu et al., 2014; Särkämö et al., 2014; Cheung et al., 2018; Tang et al., 2018). Our findings of improved performance in the specific cognitive subdomains of verbal fluency and attention are also consistent with previous research. Specifically, improvements in verbal fluency have been reported by Brotons and Koger (2000), Van de Winckel et al. (2004), Cheung et al. (2018), and Lyu et al. (2018). Särkämö et al. (2014) also found an improvement in general cognition and attention (as measured by the MMSE and Frontal Assessment Battery) after 10 weeks of either group singing or group music listening interventions. Taken together, these results suggest that a music program that involves actively applying seven crucial capacities of music is cognitively enhancing, particularly for verbal fluency and attention.

Importantly, our results also showed that the time in aged care residency predicted the degree of cognitive improvement that occurred after the MMM program. In other words, the longer a participant had spent in residency, the less global cognitive improvement they showed after MMM participation. Interestingly, *post hoc* analysis showed that by removing one participant who was an outlier with the longest time in residency, the comparison of participants’ total ACE-III scores from T1 to T2 in the MMM group became statistically significant. This suggests that early engagement with the MMM program is most beneficial. Implementation of the MMM program in people with early stages of dementia may help to optimize cognitive function and delay admission to a residential aged care facility.

One mechanism underlying the beneficial cognitive effects we observed could be neural scaffolding, facilitated by the ‘enriched environment’ that the MMM program provided. Including all seven capacities of music may have preserved the natural scaffolding process which was under threat by neurological impairment. The MMM program also incorporated activities which emphasized several ‘active’ ingredients, for example activities that were ‘novel’ or ‘challenging,’ which may lead to ‘scaffolding enhancement’ (Park and Reuter-Lorenz, 2009). These active ingredients may facilitate neural scaffolding and improve cognition by placing the individual in a state of deep engagement. A similar phenomenon has been observed in other enriching group-based activities with healthy elderly individuals that involve learning novel and challenging skills, such as quilting or digital photography (Reuter-Lorenz and Park, 2014). Thus, the MMM program, through combining the multitude of therapeutic capacities of music, may provide the enriched environment needed for compensatory scaffolding.

We propose that the cognitive benefits of the MMM program can be attributed to the relative contribution of each of the seven capacities of music, activating neural networks and providing this compensatory scaffolding. Firstly, we utilized multiple music activities (singing, playing, moving) which may maximize widespread *engagement* of frontal, parietal, temporal, limbic/paralimbic, and cerebellar regions. In particular, the cognitive effects may have been mediated through enhanced arousal, which facilitates attention and verbal fluency. Secondly, listening to *personal* music evokes *emotions* and corresponds with neural activity in subcortical and medial frontal regions (Koelsch, 2010). Simultaneously, the limbic and paralimbic regions, associated with the processing of emotions, are activated (Blood and Zatorre, 2001). Frontal regions relatively spared from degeneration in AD and may be a hub for the intersection between music, memories, and emotions (Jacobsen et al., 2015). The *physical* movement involved in the program may have increased temporal arousal, stimulating cognitive activity. Indeed, improvement in cognition in people with dementia has been found in similar studies looking at music and movement (Van de Winckel et al., 2004; Satoh et al., 2014; Shimizu et al., 2017; Cheung et al., 2018). Adding to this effect, playing rhythmical musical instruments in a *social* setting allowed individuals to *synchronize* with the music and also with each other, which may have stimulated neural activation in the prefrontal cortex and improved cognition (Shimizu et al., 2017). Lastly, the potential therapeutic benefits of the program were enhanced by the *persuasive* nature of music. Participants retained the motivation to keep attending the MMM program as they experienced it as being an enjoyable activity, demonstrated by their qualitative statements of the sessions being ‘social’ and ‘engaging.’ In this therapeutic setting, their enjoyment lead to increased motivation for participation. Thus, by involving all seven capacities of music and placing individuals in an enriched setting, the MMM program may maximize the potential of stimulating compensatory neural scaffolds.

Our finding of a decline in global cognition in the standard care group is consistent with other research findings. For example, Tang et al. (2018) reported cognitive decline in people with dementia following standard care in a nursing home facility over 12 weeks and attributed this decline to apathy. The high occurrence of social withdrawal and isolation in people with dementia may be related to higher levels of apathy, which is associated with cognitive impairment (e.g., Benoit et al., 2012). Whilst the individuals in the standard care group had the option of attending various activities (provided by the residential aged care facility), these activities did not involve all seven capacities of the MMM program. Furthermore, many individuals did not attend the activities available. Therefore, a lack of stimulation may account for the decline in their cognition. A lack of engagement in stimulating activities later in life may hasten the decline in cognition that typically arises in aging as a result of age associated disuse (Mahncke et al., 2006).

In addition to cognition, we also assessed identity, mood and motor function. Whilst music is commonly linked with identity in dementia (McDermott et al., 2014; Baird and Thompson, 2018), this is the first study, to the best of our knowledge, to undertake an empirical investigation of this issue to examine the effect of a music-based program on identity in people with dementia. We found no significant change in our identity measure (using the ‘I am/I was’ task) after participation in the MMM program. This identity measure may not have been sensitive enough to capture changes that may have been more subtle or qualitative in nature. These constructs may have been better explored via interviews, as shown in previous research in group singing for people with dementia (e.g., Camic et al., 2013; Osman et al., 2016). Furthermore, the use of observational measures might have been more sensitive in detecting symptoms of depression and altered self identity. These measures could be more reliable than the self-assessment instruments use as they do not rely on the participant to provide a direct response and thus, may be suitable for people who have communication and other cognitive difficulties associated with dementia.

To assess mood, we used a standard measure of depression specifically designed for elderly populations (GDS-SF). Unlike other similar studies that have reported improvement on this measure after participation in music activities (e.g., Guétin et al., 2009; Cooke et al., 2010), we did not find significant differences between standard care and MMM groups. We would have expected that the assessments of mood may have reflected the subjective feedback about feeling ‘happy’ and ‘elated’ after the MMM sessions. We did, however, observe a significant correlation between number of sessions attended and level of depression (total GDS score). Thus, our finding of no change in mood scores could be a dosage related finding, in that the participants who reported lowest mood at the start of the program attended the least amount of MMM sessions, meaning they did not get the full benefit of the entire program. These results also highlight how comorbidities that exist prior to the start of a therapeutic program, such as depression, may reduce session attendance; stunting the potential for rehabilitation.

Participants responded well to the program and were able to participate in all activities. Participants’ subjective responses from the booster sessions revealed that the most enjoyed and valued aspects of the program were its social, engaging, personal and emotional nature. Through the semi-formal interviews and observations during the sessions, we found that discussing personal memories, emotional reactions to songs and also moving in time with one another enabled the creation of social connections. This reinforces the value of conducting music-based interventions in a group scenario. Similarly, participants enjoyed listening to their personally chosen songs and those chosen by the other members and reminiscing. Furthermore, we deemed the visit in session 7 from the student musicians from the local conservatorium of Music an important aspect of the study that could be implemented more often in programs. This intergenerational aspect to the program could be a way to reduce stigma associated with dementia whilst also decreasing social isolation, as previously demonstrated by Harris and Caporella (2014).

### Limitations and Future Directions

We evaluated our program as a prospective cohort study, but a number of factors should be considered when interpreting the results. First, as participation was voluntary and recruitment was from a single residential care facility, our sample is only representative of individuals in aged care with an interest in music, and not necessarily representative of all individuals with dementia. Future research should examine the benefits of the MMM program to a broader sample of participants.

Second, our cohort of participants with probable dementia was a heterogeneous group that included those with a clinical diagnosis of dementia, MCI and memory disturbance. Pooling responses from a heterogenous group of individuals makes it difficult to determine how music programs can be designed to optimally benefit people with different types of dementia. On the other hand, the heterogeneous nature of our sample means that the benefits observed may be generally applicable the wider dementia population. Indeed, the majority of participants had no musical background, suggesting that anyone can participate in and receive benefits from the MMM program, even those with no prior music training.

Third, our sample size was small, primarily owing to attrition rates from illness, death, visitors, and timing of sessions after lunch when many participants were tired and often slept. That we observed a small improvement in global cognitive function following a pilot MMM program justifies future research involving a larger sample size, and a RCT design.

Fourth, it is important to consider the impact of practice effects for the measures that were used at week 2, 4, and 6 in the MMM group (but not throughout the period of standard care). The repeated measures design may have familiarized participants with test measures, thereby confounding the effects of the MMM intervention (Goldberg et al., 2015). However, the categorical verbal fluency test, which was used most frequently in the study, has been shown not to be susceptible to practice effects in people with dementia over short time intervals, such as weeks (Cooper et al., 2004). Thus, it is unlikely that practice effects can account for these specific results.

Fifth, as an initial examination of the MMM program, the research provides little insight into the optimal dosage of the intervention. Future research should evaluate the duration and dosage of such a program to potentially maximize the cognitive benefits of the MMM program. Although visiting each participant on a weekly basis may not be feasible by residential aged care facility staff, the improvement in cognition in our results indicated that potentially one full group MMM session plus an individual booster MMM session per week is valuable. Similarly, it would be worthwhile exploring whether the observed benefits of the MMM program for verbal fluency and attention might generalize to an improvement in communication, social skills or activities of daily living.

Sixth, although the MMM program is based on a model of the active ingredients (capacities) of music-based interventions, the current investigation does not provide a full understanding of the specific benefits of each capacity and its therapeutic outcome, as seen in the TMCM. Future research could include comparisons of the full MMM program with programs that focus on more specific sets of capacities, such as music and reminiscence (*emotional* and *personal*) or music and movement (*physical* and *synchronous*). Further research could also compare the MMM with other arts-based programs to determine whether music is unique in its effects, or whether any social activity can lead to benefits (as seen in Narme et al., 2014 who found no short-term behavioral differences between music and cooking).

Finally, the MMM program could be trialed with people with other neurological disorders such as Parkinson’s disease or stroke. In our cohort of participants, we had two individuals with Parkinson’s disease and one with post stroke aphasia. These individuals were capable of participating in all activities in the MMM program to the same extent as the participants with dementia.

## Conclusion

We have devised and evaluated a music-based treatment that is grounded in a model of the therapeutic application of music, the TMCM. A primary advantage of the MMM program is that activities are linked with plausible mechanisms of intervention. This investigation not only furthers our understanding of music-based treatments but, unlike other protocols, addresses individual goals. Therapists can emphasize specific activities that target specific problems faced by individuals with neurological disorders. Our results demonstrate that the MMM program may benefit cognition, particularly attention and verbal fluency, in people with various types of dementia. By including all seven capacities of music in one program, individuals were placed in a particularly enriched environment likely to promote neural scaffolding. Our findings highlight the importance of engaging people in such programs soon after admission to a residential aged care facility (or even earlier) to gain optimal cognitive benefits. The small and heterogenous sample limits the conclusions that can be drawn, and underscores the need for additional research to be conducted on these effects. Nevertheless, our findings encourage the development and refinement of music-based interventions that build on evidence and theory, and that may be personalized to suit the various needs of people with dementia.

## Ethics Statement

The application was considered by the Macquarie University Human Research Ethics Committee [HREC (Medical Sciences)]. Reference No: 5201700523. Initial contact was made to the participant by aged care facility staff that they are familiar with. If the participant was willing, the aged care facility staff then communicated the suitability of participants to the investigators. Consent for participation and review of medical records was obtained from the significant other of potential participants and participants themselves. Given the mobile fragility of many of the participants they were asked to only move within their limits. Any emotional distress was dealt with by having contact with the staff at the care facility who knew the participants mental state well. Before the beginning of each session participants were assessed as to whether they were well enough to participate.

## Author Contributions

OB and AB coordinated testing and data collection. OB was responsible for data analysis and all authors contributed to data interpretation. OB wrote the first draft of the manuscript and all authors contributed to further revisions. All authors approved the final version of the manuscript and contributed to the design and development of the study.

## Conflict of Interest Statement

The authors declare that the research was conducted in the absence of any commercial or financial relationships that could be construed as a potential conflict of interest.

## Appendix A:

### List of Songs

1. Glen Miller – In the Mood
2. Glen Miller – Chattanooga Choo Choo
3. Judy Garland – Somewhere Over the Rainbow
4. Gene Kelly – Singing in the Rain
5. Nat King Cole – Too Young
6. Doris Day – Que Sera Sera
7. Doris Day – Black Hills
8. Dinah Shore – Buttons and Bows
9. Bing Crosby and The Andrews Sisters – Don’t Fence Me In
10. Bing Crosby – Swing on a star
11. Bing Crosby – White Christmas
12. Perry Como – Catch a falling star
13. Frank Sinatra – Come fly with me
14. Little Eva – The Locomotion
15. Buddy Holly – Peggie Sue
16. Sam Cooke – Cupid
17. Ella Fitzgerald – A-Tisket, A-Tasket
18. Bill Haley and his Comets – Joey’s song
19. Elvis – It’s now or never
20. The Beatles – Hey Jude
21. Some Enchanted Evening
22. My Bonnie lies over the ocean
23. Oklahoma ‘Oh what a beautiful morning’
